# The efficacy and safety of intra-articular injection of corticosteroids in multimodal analgesic cocktails in total knee arthroplasty—a historically controlled study

**DOI:** 10.3389/fsurg.2024.1279462

**Published:** 2024-06-11

**Authors:** Yixiong Wang, Zhichang Li, Xuan Gao, Jianhao Lin

**Affiliations:** ^1^ Department of Orthopedic Diseases, Jincheng General Hospital, Jincheng, China; ^2^ Arthritis Clinic & Research Center, Peking University People’s Hospital, Beijing, China; ^3^ Department of Orthopedic Disease, Luanzhou People’s Hospital, Tangshan, Hebei, China

**Keywords:** total knee arthroplasty, analgesia, intra-articular injection, corticosteroid, efficacy, safety

## Abstract

**Background:**

Total knee arthroplasty (TKA) is a common and effective procedure. Optimizing pain control and reducing postoperative discomfort are essential for patient satisfaction. No studies have examined the safety and efficacy of intra-articular corticosteroid injections following TKA. This study aims to examine the safety and efficacy of corticosteroids in intra-articular multimodal analgesic injections.

**Materials and methods:**

This was a historically controlled study conducted at a single academic institution. Before May 2019, patients received an intra-articular cocktail injection without corticosteroids during surgery, referred to as the non-corticosteroid (NC) group. After June 2019, intraoperatively, patients received an intra-articular cocktail injection containing corticosteroids, referred to as the corticosteroid (C) group. Finally, 738 patients were evaluated, 370 in the C cohort and 368 in the NC cohort. The mean follow-up duration was 30.4 months for the C group and 48.4 months for the NC group.

**Results:**

The mean VAS scores at rest on postoperative day (POD) 1 (2.35) and POD3 (3.88) were significantly lower in the C group than those in the NC group, which were 2.86 (POD1) and 5.26 (POD3) (*p* < 0.05). Walking pain in the C group (4.42) was also significantly lower than that (5.96) in the NC group on POD3 (*p* < 0.05). Patients in the C group had a significantly higher mean range of motion (ROM) (92.55) on POD3 than that (86.38) in the NC group. The mean time to straight leg raise for group C (2.77) was significantly shorter than that (3.61) for the NC group (*p* < 0.05). The C group also had significantly fewer rescue morphine (1.9) and metoclopramide (0.21) uses per patient than the NC group, which were 3.1 and 0.24, respectively. No significant differences in fever or vomiting rates between groups were found. Patients in neither group developed periprosthetic joint infections or skin necrosis. One patient in the C group suffered from wound dehiscence, and the wound healed well after debridement. No patient died or had a re-operation in either group.

**Conclusions:**

This pilot trial found that intra-articular injection of multimodal analgesia (including corticosteroids) reduced initial postoperative pain, increased ROM in the early postoperative days (up to POD3), and did not increase wound complications or infection rates in approximately 30 months of follow-up.

## Introduction

Total knee arthroplasty (TKA) is a frequent and highly effective procedure. It reduces pain and improves knee function in individuals with end-stage knee osteoarthritis, (1). Nonetheless, total joint arthroplasty (TJA) is an intrinsically painful surgical treatment. Considering the rising demand for arthroplasty, there is a growing necessity for standardized multimodal analgesic regimens. Optimizing pain control and decreasing postoperative discomfort for patients having TJA are not only essential for patient wellbeing but are also becoming increasingly relevant in value-based patient care (2). Appropriate pain management has been associated with accelerated rehabilitation and enhanced patient satisfaction measures, including health-related quality-adjusted life years, return to work, and overall satisfaction (2, 3).

Multimodal pain management, introduced by Kehlet and Dahl in 1993 (4), is an integral part of perioperative management. The term refers to using multiple agents that target different sections of the nociception pathway. The objective is to promote better pain management with reduced dependency on opioids, hence minimizing the associated opiate adverse effects.

By adding steroids to perioperative drug protocols, which is called steroid supplementation, either through intravenous (IV) administration (5) or periarticular infiltration (PAI) (5), pain, opioid use, nausea and vomiting, limited range of motion, and inflammatory markers can be reduced without increasing short- and mid-term complications (6).

Intra-articular injection could be a faster approach than PAI. In this strategy, corticosteroids could be mixed with other frequently used drugs in multimodal analgesics (cocktails), such as long-acting local anesthetics, epinephrine, and the like. According to the author's knowledge, no published research has investigated the efficacy and safety of intra-articular injections of corticosteroid-containing cocktails after TKA. The objective of this study is to evaluate the efficacy and safety of adding corticosteroids to the intra-articular injection of multimodal analgesics. In the early postoperative phase, we expected that the addition of 4 mg of Diprospan (compound betamethasone injection) to cocktail solutions, including ropivacaine, epinephrine, and morphine, diluted to 50 ml, would result in improved pain control, ROM recovery, and decreased nausea and vomiting. In addition, we anticipated that the use of Diprospan (containing 5 mg of betamethasone dipropionate + 2 mg of betamethasone sodium phosphate) does not increase the rate of periprosthetic joint infection (PJI) in the short-term follow-up after TKA.

## Materials and methods

### Study design

This was a historically controlled study conducted at a single academic institution, comparing intra-articular injections of analgesic cocktails with or without corticosteroids for patients undergoing TKA from January 2018 to August 2020. Before May 2019, patients received intra-articular cocktail injections without corticosteroids, referred to as the non-corticosteroid (NC) group. After June 2019, patients received intra-articular cocktail injections containing corticosteroids, referred to as the corticosteroid (C) group. The study was approved by the institutional review board (registration no. R0019) and was completed in accordance with the Declaration of Helsinki.

### Participants

A retrospective review of institutional medical records was conducted. Patients with end-stage knee osteoarthritis who received unilateral primary TKA under spinal anesthesia and had a length of stay >7 postoperative days (POD) to characterize postoperative opioid consumption and pain scores more accurately were assessed for eligibility. Inclusion criteria were the following: (1) age between 50 and 80 years; (2) falling under Classes I–II of the American Society of Anesthesiologists (ASA) physical status classification; (3) patients who received an intra-articular injection of multimodal cocktail, with or without corticosteroids; and (4) patients who received both the sciatic nerve and femoral regional nerve blocks. Exclusion criteria were the following: (1) allergic and/or contraindications to the medications used in the study; (2) severe lumbar radiculopathy that leads to obvious sensory motor deficits; (3) cognitive impairments and/or inability to understand the study protocol, and/or refusal to consent; (4) uncontrolled diabetes mellitus (HbA1C greater than 8.0); (5) previous knee infection and/or surgery other than arthroscopic meniscectomy; (6) chronic systematic usage of corticosteroids because of inflammatory and/or autoimmune diseases; (7) intraoperative corticosteroids administered by the anesthesiologist; and (8) contraindications to spinal anesthesia.

**Figure 1:**
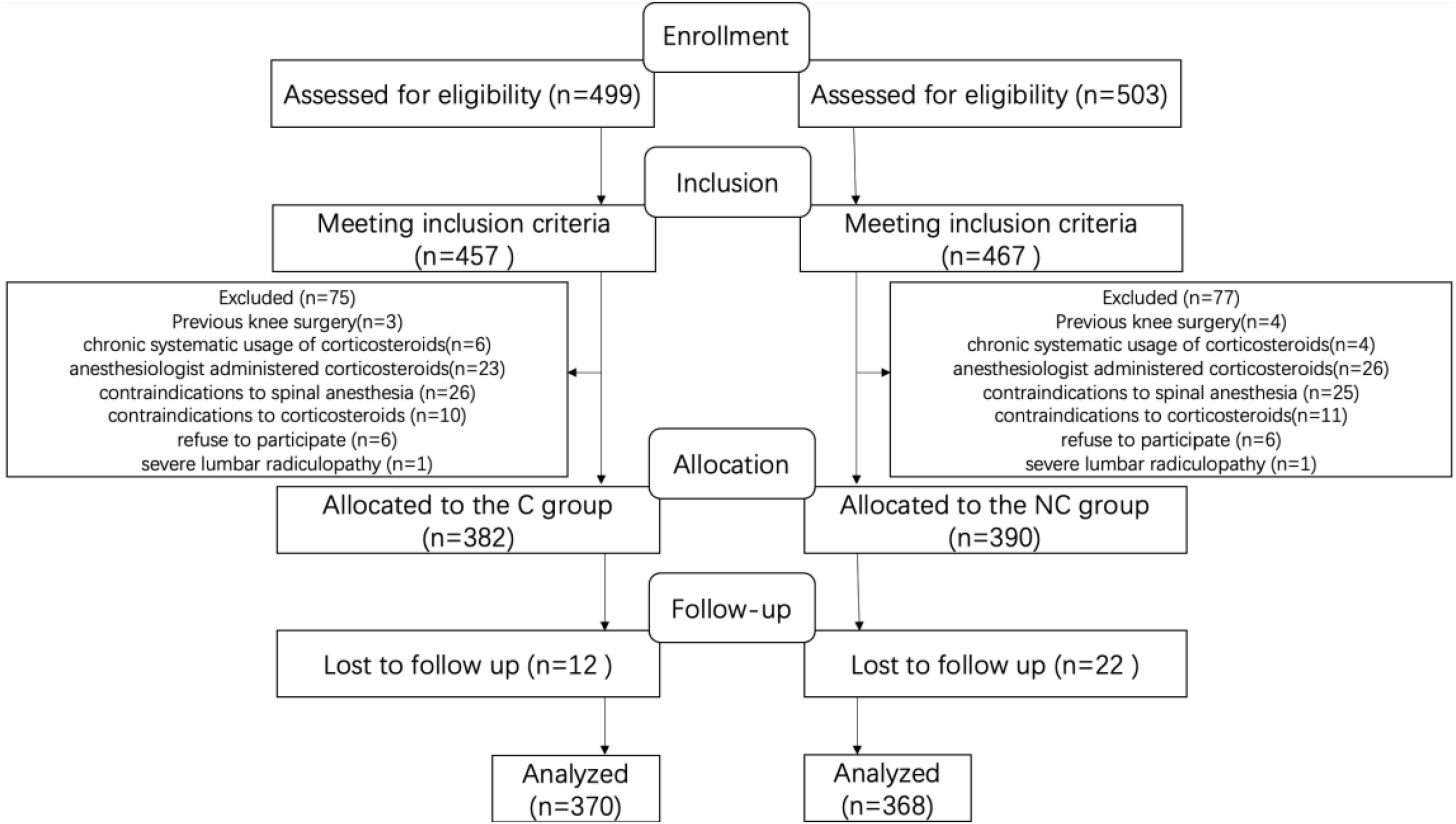
Diagram outlining the flow of patients within the present study.

In the study period, 1,023 patients with end-stage knee osteoarthritis who received unilateral primary TKA under spinal anesthesia were identified. Also, 21 patients were discharged home before POD 7. These 21 patients were not assessed for eligibility. As shown in Figure 1, 1,002 patients received TKA under spinal anesthesia, of whom 924 were included. A total of 772 patients remained after exclusions. Reasons for exclusions are provided in Figure 1. Thirty-four patients were lost to follow-up. The final number of patients analyzed totaled 738, with 370 in the C cohort and 368 in the NC cohort.

### Interventions

In both groups, the intra-articular injection contained 10 ml of 7.5 mg/ml ropivacaine; 5 ml of 10 mg/ml morphine hydrochloride hydrate, 2 drops of 1 mg/ml adrenaline, and 20 ml of 50 mg/ml tranexamic acid (TxA). In the C group, the analgesic cocktails also contained one injection of compound betamethasone (containing 5 mg of betamethasone dipropionate + 2 mg of betamethasone sodium phosphate), while in the NC group, the cocktails did not contain steroids. The cocktail solutions were diluted to 50 ml solutions with 0.9% saline in both groups.

### Surgery and perioperative management

Patients received femoral and sciatic nerve blocks during anesthesia preparation. At the induction of anesthesia, all patients received standardized antibiotic prophylaxis (1.5 g of cefuroxime) and 1 g of TxA intravenously.

The surgeries were performed by the senior surgeon (ZL). Unilateral primary TKA was performed in all patients using either the Depuy Attune Cruciate Retaining (CR) prosthesis or Biomet Vanguard CR prosthesis with the measured resection technique using mechanical alignment. All surgeries were performed with a standard midline incision and a medial parapatellar arthrotomy. An intramedullary femoral guide and an extramedullary tibial guide were used in all cases. The patella was selectively resurfaced. All surgeries were performed with short-duration tourniquet use, with the tourniquet inflated just before cement application and deflated after its hardening, and all components were cemented in this series. After cementation, the tourniquet was released, and hemostasis was achieved before closing the joint. With the knee in flexion, wounds were sutured in layers. After closing the joint capsule, the cocktail solution was injected intra-articularly, either with or with betamethasone. Drain was used, clamped for 6 h, and removed at 24 h postoperatively.

Right after the surgery, patients were routinely given a single dose of ondansetron to prevent nausea and vomiting and were given ice packs to the operated knees three times a day during their hospital stay. Since POD1, patients received regular meloxicam at 7.5 mg twice daily and aminophenol oxycodone at 5 mg twice daily. If a patient was unable to tolerate the pain, a rescue analgesic (10 mg of morphine hydrochloride) was injected subcutaneously. For patients experiencing vomiting, metoclopramide at 5 mg was given orally.

Early rapid mobilization was facilitated for physical prophylaxis of thrombosis. Since POD1, patients received aspirin at 325 mg twice daily for 30 days. They were also provided with below-knee antiembolism compression stockings in the postoperative period for 2 weeks.

All patients started range of motion without restriction since POD1 and were encouraged to stand with support on POD2 after the nerve blocks were removed.

Patients were referred to the rehabilitation center on POD4 or POD5, provided they were medically stable, where they underwent knee ROM exercise and muscle strength training. If no complications occurred, patients were discharged home on POD14 or 15, after the sutures were removed.

### Outcome measurements

Preoperative patient characteristics were obtained for all patients, including age, sex, ASA score, etc.

The primary outcomes included postoperative pain, ROM, and the day the patient achieved straight leg raising. Postoperative pain at rest and during walking at different time points (POD1, 3, 5, 7, 42) was assessed using the visual analog scale (VAS) from “no pain” (0 point) to “extreme pain” (10 points). The worst pain experienced by the patient at rest and during walking was recorded. Active ROM was measured with a goniometer on POD3 and POD7.

Secondary outcome measures included the following: (1) the number of rescue analgesia used in the first operative 7 days; (2) The number of the uses of metoclopramide in the first operative 7 days; (3) the number of patients who had a fever; and (4) the number of patients who experienced vomiting.

Postoperative complications, such as PJI and wound complications, were also recorded. Patients were assessed for any local (hypersensitivity reaction, PJI, wound dehiscence, bleeding, or tendon rupture) or systemic (cardiovascular issues, renal impairment, stroke) adverse events of corticosteroids.

In the outpatient clinic on POD42, patients were assessed for postoperative conditions, including rest and walking pain, active knee ROM, and any potential complications. During the last follow-up, patients were assessed via phone for any signs of PJI. Patients were asked about complaints related to the operated knee. In case of any knee complaints, including knee pain, recalcitrant swelling, and/or limited ROM (active flexion less than 90°), patients were instructed to undergo blood tests including erythrocyte sedimentation rate (ESR) and C-reactive protein (CRP). In case of any elevated ESR and/or CRP, patients were advised to undergo arthrocentesis to test for the presence of PJI.

### Sample size calculation

We considered a one-point decrease in VAS score as the minimal clinically important difference based on previous studies on pain associated with corticosteroid use in TKA (7). Power analysis indicated a minimum sample size of 76 for each trial arm [standard deviation (SD) 23, *α* = 0.05, *β* = 0.80]. We decided on a minimum sample size of 85, assuming a dropout rate of 10%.

### Statistical analyses

Continuous data were compared using paired *t*-tests, while categorical and discrete data were compared using a chi-squared test between the two groups. All tests were two-sided, and *p* < 0.05 was considered statistically significant. All analyses were performed using SPSS (IBM SPSS Statistics, version 20).

## Results

### Enrollment and follow-up

A total of 370 of 382 patients (96.9%) in the C cohort group and 368 of 390 patients (94.4%) in the NC cohort completed the last follow-up assessment. A total of 738 patients were included in the analysis. The mean follow-up time was 30.4 months in the C group and 48.4 months in the NC group. The minimum follow-up time was 40.2 months in the C group and 25.6 months in the NC group.

### Patient characteristics

There were no significant differences in patients’ baseline characteristics in terms of demographic factors, ASA classifications, preoperative knee pain, ROM, preoperative laboratory parameters, and surgical factors (Table 1).

**Table 1:** Patient demographics.

	C (370)	NC (368)	*P*-value
Age (years)	67.1	65.6	0.173
Gender (M/F)	114/256	111/257	
Height (cm)	161.4	163.9	0.077
Weight (kg)	70.4	69.6	0.057
Operative time (min)	71.8	71.5	0.412
ASA	I: 160	I: 165	
	II: 210	II: 203	** **
Diabetes mellitus	13.2% (49)	12.5% (46)	
Preoperative ROM	93.1	95.6	0.226
Tourniquet time (min)	14.8	14.9	0.652
ESR	14.8	14.6	
CRP	4.3	4.5	
Preoperative rest pain	6.15	6.00	0.311
Preoperative walking pain	6.8	6.8	0.368
Prosthesis used	370	368	
Biomet Vanguard fixed bearing CR	130	125	
Depuy Attune mobile bearing CR	144	148	
Depuy Attune fixed bearing CR	96	95	

### Pain, ROM, and the time when patients achieved straight leg raising

Outcome comparisons on pain, ROM, and the time when patients achieved straight leg raising are presented in Tables 2 and 3. Patients in the C group had significantly less rest pain on POD1 and POD3 and less walking pain on POD3. Also, patients in the C group had significantly greater ROM on POD3. In addition, the day when patients achieved straight leg raising in the C group was significantly earlier than that in the NC group. There were no significant differences between groups in any other comparisons.

**Table 2:** Outcome comparisons on pain.

	C	NC	*P*-value
Preoperative rest pain	6.15	6.00	0.311
Rest pain on POD1	2.35	2.86	0.018*
Rest pain on POD3	3.88	5.26	0.000*
Rest pain on POD5	3.53	3.61	0.096
Rest pain on POD7	2.66	2.72	0.322
Rest pain on POD42	1.92	1.93	0.898
Preoperative ROM	95.1	95.6	0.426
Preoperative walking pain	6.80	6.76	0.368
Walking pain on POD3	4.42	5.96	0.008*
Walking pain on POD5	4.31	4.44	0.106
Walking pain on POD7	3.98	4.11	0.350
Walking pain on POD42	2.11	2.21	0.672

*P < 0.05.

**Table 3:** Outcome comparisons on ROM.

	C	NC	*P*-value
Preoperative ROM	94.14	95.62	0.226
ROM on POD3	92.55	86.38	0.001*
ROM on POD7	96.39	95.33	0.103
ROM on POD42	117.29	116.46	0.757
Straight leg raising	2.77	3.27	0.010*

*P < 0.05.

### Discomfort and the incidence of fever and vomiting

The numbers of uses of rescue morphine consumption (1.9, range 0–4, SD 1.6) and metoclopramide (0.21, range 0–2, SD 0.35) per patient were also significantly lower in the C group (Table 4) than those (morphine: 3.1, range 0–6, SD 2.9; metoclopramide: 0.24, range 0–3, SD 0.38) in the NC group. The percentages of patients with a fever or vomiting were not significantly different between groups (Table 4).

**Table 4:** Number of the use of rescue analgesia and metoclopramide during the first 7 postoperative days and the incidence of fever and vomiting.

	C	NC	*P*-value
Mean number of morphine consumption per patient	1.9	3.1	0.013*
Percentage of patients who had a fever	4%	5%	0.383
Mean number of uses of metoclopramide per patient	0.21	0.24	0.048*
Percentage of patients who experienced vomiting	10%	11%	0.532

*P < 0.05.

### Safety and complications

Complications were shown in Table 5. Patients in neither group developed PJI or skin necrosis. One patient in the C group suffered from wound dehiscence, but the wound healed well after a surgical debridement. No patient died or had a re-operation or revision in either group.

**Table 5:** Outcome comparisons on complications.

	C (370)	NC (368)	*P*-value
PJI	0	0	NA
Wound dehiscence	1	0	1.000 (Fisher exact two-sided)
Skin necrosis	0	0	NA

## Discussion

This study found that intra-articular injection of multimodal analgesia (including corticosteroids) lowers pain, increases ROM in the acute postoperative phase (up to POD3), and does not increase wound complications or infection rates in approximately 30 months of follow-up. To the author's knowledge, this is the first study to investigate the efficacy and safety of intra-articular corticosteroid injection as a component of multimodal pain management in TKA. Prior research has concentrated on IV and periarticular injections as the primary route of steroid administration. Overall, excellent clinical outcomes were observed.

According to the findings of a recent systematic review, adding a corticosteroid to the periarticular injections further reduces postoperative pain and may minimize postoperative opioid intake (5). In another meta-analysis with 829 patients, Li et al. reported that the addition of a corticosteroid to periarticular injections improved daily postoperative pain scores until day 3 and decreased postoperative opioid consumption (8). In the current study, we found that patients who received analgesics containing corticosteroids had significantly reduced rest pain on POD1 and POD3 and reduced walking pain on POD3 and POD5.

It is important to note that the original research included in that meta-analysis (8) yielded inconsistent results. Four randomized controlled trials (RCTs) found that adding corticosteroids to periarticular injections reduced postoperative discomfort (7, 9–11). Nonetheless, in the other three RCTs, there was no difference in postoperative pain among individuals who received periarticular injections with or without corticosteroids (12–14). Sean et al. discovered that adding triamcinolone to the injection reduced cumulative postoperative opioid consumption compared to ropivacaine alone in their study of 100 primary TKA patients (10). In contrast, the other four out of the five studies that reported postoperative opioid intake following primary TKA with periarticular corticosteroid injection were not statistically different compared to the control group (15).

In terms of function, a systematic review of RCTs found that patients who received corticosteroid infiltration exhibited a transitory increase in ROM on postoperative days 1, 2, and 3 (16). In the current study, we also found significantly greater ROM on POD3 but not on POD7. In the current study, the corticosteroid used was the compound betamethasone, whose plasma biological half-life (100–300 min) is comparable to that of dexamethasone. Another systematic review of RCTs showed that the use of periarticular injections with corticosteroids has decreased the time required to achieve straight leg raise (17), which was consistent with the current study. In the systematic review, the length of hospital stay and proinflammatory signals were also decreased in patients with steroid administration (17).

In a retrospective observational study, data from 435 patients who received periarticular injections using ropivacaine with or without dexamethasone were reviewed. The overall incidence of postoperative nausea and vomiting was 23.2%. The incidence of postoperative nausea and vomiting within 24 h was lower in patients who received periarticular injections with dexamethasone than in those who received periarticular injections without dexamethasone (19.5% vs. 49.1%, *P* < 0.001) (18). In the current study, there was a significant but subtle difference in the use of metoclopramide per patient between the C (0.21) and the NC (0.24) groups. Nonetheless, there was no difference between the percentages of patients who experienced vomiting.

As for adverse events, in a meta-analysis recently published by AAHKS (5) that included eight high-quality RCTs, five reported complications (7, 9, 12, 13, 15). Four of the five studies reported no differences in the incidence of PJI and wound complications between groups with or without the addition of corticosteroids to periarticular injections. Only one study documented a solitary instance of PJI in the group administered high doses of corticosteroids. Following that, the patient underwent a two-stage revision arthroplasty (13). However, all studies had insufficient sample sizes (7, 9, 12, 13, 15) to draw conclusive conclusions regarding the ratio of complications, such as wound complications and surgical site infections, due to their infrequent occurrence.

Similar results were reported in articles focusing on IV administration of corticosteroids. Strong evidence supports the use of a single or multiple doses of intravenous dexamethasone to reduce postoperative pain, opioid consumption, nausea, and vomiting after primary TJA (5, 6); In addition, IV corticosteroids are effective in decreasing ROM limitation (6) and are not associated with increased risks of complications (5). Compared to periarticular injections, IV administration of corticosteroids yields better results, especially in controlling postoperative nausea and vomiting, as the antiemetic effect is related to systemic absorption and plasma concentration of dexamethasone (19).

Numerous studies have focused on the mechanisms of corticosteroids regarding their analgesic and antiemetic effects. There is a close relationship between postoperative pain and inflammatory response (20). Some studies have shown that corticosteroids can alleviate pain by inhibiting the inflammatory response (21, 22). The corticosteroids act by inhibiting phospholipase A2 and reducing production of cyclooxygenase and lipoxygenase pathway products, leading to decreased mediators of systemic inflammation, pain, and acute stress response (23). They also decrease postoperative nausea and vomiting through their central antiemetic effect by inhibiting prostaglandin production and endogenous opioid release. Saini et al. verified that the anti-inflammatory effect of periarticular injection of 8 mg of dexamethasone was comparable to that of IV injection of 8 mg of dexamethasone in the initial 24 h following surgery. However, the antiemetic effect in the periarticular injection group was comparatively weaker than that in the IV group, potentially due to its reduced systemic absorption (19).

According to previous publications, neither periarticular injections nor IV administration of corticosteroids is associated with increased septic complications. Nonetheless, previous studies have suggested that preoperative intra-articular corticosteroid injections increase the incidence of PJI in subsequent TKA. This may partially be the reason why surgeons and or investigators have not used intra-articular corticosteroid injections as an adjunct to postoperative analgesic protocols previously. Data from 173,465 arthroplasties in the hip or knee, as well as from 73,049 injections and 100,416 control patients (24), suggest that ipsilateral intra-articular corticosteroid injections within 3 months before arthroplasty were associated with an increased risk [OR 1.39 (95% CI 1.04–1.87); *p* = 0.03] of PJI during subsequent joint arthroplasty (24).

Thus, there were conflicting results on whether corticosteroid application increases the incidence of PJI. Preoperative intra-articular injections of corticosteroids increased the incidence of PJI, while perioperative or intraoperative applications, either intravenously or via periarticular injections, did not increase the incidence of PJI, according to the best available evidence.

We consider that important reasons for this discrepancy might include preoperative optimization of patient risk factors (25), surgical site antisepsis (26), extensive irrigation (27), and prophylactic application of antibiotics (28).

Moreover, in an *in vitro* study, Artini et al. analyzed the *in vitro* effect of a commercial formulation of betamethasone (Bentelan) on several Gram-positive and Gram-negative bacteria of clinical relevance. It was found to be an inhibitor of the growth of most of the strains examined. Also, the effect of betamethasone in combination with some classes of antibiotics was evaluated. Antibiotic–steroid combination therapy is, in such cases, superior to antibiotic-alone treatment in impairing bacterial growth. Nonetheless, such effect was essentially not at all observable in *Staphylococcus aureus* or coagulase-negative staphylococci (CoNS) (29).

We postulate that the biological responses after intra-articular injections and periarticular injections of corticosteroids would be similar. As of now, no PJI cases have been observed by far in this cohort of patients through at least 2 years of follow-up. Nevertheless, we should be very conservative that the sample size in this study might not be sufficient enough to test the incidence of PJI, which is not frequent. In the current study, there was a single case of surgical site infection in the corticosteroid group. The patient suffered from wound dehiscence, which was treated successfully by surgical debridement. Statistically, there is no difference in the number of patients in our study. In contrast, we cannot disprove that corticosteroids played a role in this negative outcome.

The limitation of the current study included, first, its retrospective nature. The data were retrieved according to the institutional medical record database. Second, the sample size was powered based on the VAS pain score but was probably underpowered for the ratio of complications, including surgical site infection and wound complication, owing to their low frequency. Third, this study was conducted in a single center with involvement from two surgeons. All patients underwent TKA with spinal hypotensive anesthesia and received sciatic nerve and femoral nerve blocks, and the corticosteroid used in the current study was betamethasone. The preoperative rest pain was comparatively more severe than those in other similar studies. Also, the length of hospitalization stay was usually longer than that in other studies and settings. This design ensured that the data on POD5 and POD7 were accurate. However, the results generated from the current study may not be generalizable to patients receiving TKA with less preoperative pain, with other settings, other anesthetic regimens, and other selections of corticosteroids. Fourth, in the current study, the circumstances of chronic opioid users were not understood, which is a confounding factor, as patients who chronically use opioid medications prior to total knee arthroplasty may be at a substantially greater risk for complications and painful prolonged recoveries (30). Further prospective multicenter RCTs with a greater number of patients and a longer follow-up period are required to determine the generalizability of our findings, especially the efficacy of the procedure. Observational studies with large sample sizes are required to test the safety of the procedure. Also, further investigation is needed in patients managed under other settings, anesthetic techniques, and medication regimens.

The injection site in the periarticular injections could be a posterior capsule, quadriceps tendon, intra-articular synovium, or a combination of the above anatomic sites, which varies from different studies and could impact the efficacy of the periarticular corticosteroid injections. Also, there is a lack of an accepted consensus on injection sites. Compared to periarticular injections, the intra-articular approach is inherently technically easier and more time saving. After the flexion and extension of the knee, the analgesic drug needs to be evenly distributed in the joint. In addition, the procedure of injection also checks the waterproofness of the joint capsule suture.

## Conclusion

The current study is pilot research investigating the effectiveness and safety of intra-articular injection of steroids as an integral part of the multimodal analgesics in TKA. Future studies comparing different approaches, including intra-articular and periarticular injections and IV administration, are also warranted to determine the best route and dose of corticosteroid administration.

## Data Availability

The original contributions presented in the study are included in the article/Supplementary Material, further inquiries can be directed to the corresponding author.
